# Exploring IoT Location Information to Perform Point of Interest Recommendation Engine: Traveling to a New Geographical Region

**DOI:** 10.3390/s19050992

**Published:** 2019-02-26

**Authors:** Xu Yang, Billy Zimba, Tingting Qiao, Keyan Gao, Xiaoya Chen

**Affiliations:** School of Computer Science and Technology, Beijing Institute of Technology, Beijing 100081, China; zimba.billy@gmail.com (B.Z.); 3220180845@bit.edu.cn (T.Q.); 3220180795@bit.edu.cn (K.G.); 3220180788@bit.edu.cn (X.C.)

**Keywords:** data science, personalized recommendation, location sensor, point-of-interest, internet of everything

## Abstract

With the development of wireless Internet and the popularity of location sensors in mobile phones, the coupling degree between social networks and location sensor information is increasing. Many studies in the Location-Based Social Network (LBSN) domain have begun to use social media and location sensing information to implement personalized Points-of-interests (POI) recommendations. However, this approach may fall short when a user moves to a new district or city where they have little or no activity history and social network friend information. Thus, a need to reconsider how we model the factors influencing a user’s preferences in new geographical regions in order to make personalized and relevant recommendation. A POI in LBSNs is semantically enriched with annotations such as place categories, tags, tips or user reviews which implies knowledge about the nature of the place as well as a visiting person’s interests. This provides us with opportunities to better understand the patterns in users’ interests and activities by exploiting the annotations which will continue to be useful even when a user moves to unfamiliar places. In this research, we proposed a location-aware POI recommendation system that models user preferences mainly based on user reviews, which shows the nature of activities that a user finds interesting. Using this information from users’ location history, we predict user ratings by harnessing the information present in review text as well as consider social influence from similar user set formed based on matching category preferences and similar reviews. We use real data sets partitioned by city provided by Yelp, to compare the accuracy of our proposed method against some baseline POI recommendation algorithms. Experimental results show that our algorithm achieves a better accuracy.

## 1. Introduction

The rapid growth of cities has led to an increase in the number of points of interest (POIs), e.g., restaurants, theaters, stores, hotels, to enrich people’s life and entertainment, providing us with more choices of life experience than ever before. People are willing to explore the city and their neighborhood in their daily life and decide which places to go according to their interest and various choices of POIs. However, under the big data scenario, because of the huge number of possible POIs across cities and complexity of modern cities as well as unfamiliarity to new individuals in these cities, finding a POI or making a satisfactory decision efficiently among the large number of POIs becomes a problem for people. Fortunately, with the rapid pervasiveness of mobile devices embedded with wireless communication and location sensors, they form into a internet of everything. Location-based social networks (LBSNs) mobile applications such as Foursquare, Yelp, and Facebook, have become some of the most popular Internet applications and attracted millions of users as they help solve the problem of finding places in a specific physical geographical area for users. Through these applications, across the world, individuals share their footprints, opinions, experiences and contribute assorted forms of location-specific multimedia contents by declaring their presence by an action known as a check-in which is very helpful for individuals wishing to find a new restaurants, events, bars, and etcetera.

However, even with the availability of information generally presented by LBSNs a user will still be subjected to massive information in most cases biased on popularity of a POI rather than individual preference. More specifically to this study, a user traveling or migrating to a new geographical region with no prior information about the new geographical region will have a hard time deciding which POI to visit aside from the tourist attractions. Thus, a need to make relevant recommendations according to the individual user making the query. Indeed, a study of human interactions and collective user behaviors on an unprecedented scale i.e., across different geographical regions, will yield fruitful results in delivering point of interests suggestion relevant to a user according to his/her preferences irrespective of whether they are new to the area or old.

Recommendation systems are software tools and techniques that provide suggestions for items to be of use to a user. In this paper, we refer to the recommendation systems that provides suggestion for POIs from vast amount of available POIs in a huge city to assists with decision making processes such as which restaurant to go to for dinner, which museum to be visited, and many more.

Many researchers and industrial specialist have done a significant amount of work in studying and analyzing such structures in order to facilitate a user’s exploration and decision making by POI recommendation. Extensive knowledge about an individual’s interests and location-oriented behaviors can be learned from his/her geographically bound activities in LBSNs, which makes POI prediction or ranking for recommendation in LBSNs challenging, interesting and different from that in non-geographical based online social networks. With the availability of such information in LBSNs, an intuitive idea for supporting POI recommendations is to employ the conventional collaborative Filtering (CF) [1,2,3,4] techniques by treating POIs as the "items" in many successful CF-based recommender systems. A technique based on the knowledge that if users show similar behavior in the past, they will continue to do so in the future (users or friends with a similar geographical footprint).

Through this approach, incorporating social influence has been shown to be successful, as it forms a foundation for POIs recommendation system integrated with additional content-based information or influences such as geographical proximity/patterns, user information, venue information, temporal dynamics etc. A recent enhancement that has been added to make these systems more personalized hence accurate is modeling of a user’s personal geographical footprint for example, indoorsy persons like visiting locations around their living areas while outdoorsy persons prefer exploring new interesting places by traveling around the world [5,6]. Moreover, recently studies have consider social influence given its strong influence as the core of their algorithm onto which other factors such as category information [7], venue information [8] have been integrated to improve the accuracy by making the recommendation more personalized.

As we filter POI to provide better matches for users from the largely available POIs, unique properties of location-based social networks come with three major challenges. These are location context awareness, heterogeneous domain and rate at which data grows. In this paper we focused on location context awareness.

Geographical dimension in LBSNs bridges the gap between the physical world and the virtual online world. Therefore, the user’s current location is very important in making suggestion as people are mobile individuals so it is critical that we provide suggestion within a user’s current geographical area. Hence, in as much as the aforementioned recommendation approach yields accurate results this challenges is non-trivial and may lead to out of context suggestion. Indeed, a location possesses a spatial constraint for providing POI recommendations, at the same time influences user preferences. Nevertheless, if a user travels to a new geographical region this becomes even more challenging because a user will have little to no historical information in this geographical area. In this case the recommendation system faces a cold start problem on such users even though a user has location history from other geographical regions.

A Cold Start problem occurs when the recommendation system encounter some individual users with very few history or activity. For the new user, the recommendation model does not have enough knowledge to provide effective suggestion. This being the case with a user in a new region with no social friends (social links) or location history in this region. This is because the new geographical region will different POIs and landscape such that the previous experiences obtain cannot explicitly interpret a user’s local preference in the new region. In addition, social links which have been based on POIs overlaps or friendship may not apply as a particular user is not guaranteed overlapping links in this new environment. For example, beaches might be given a high recommendation score to a user traveling to seaside city, even though the user prefers sporting events more than beaches typically. The same user may be more interested in seeing the status of her friends living in this city. Thus, it is much better to devise a new and sophisticated method to exploit the characteristics of LBSNs, social influence, category, temporal dynamics and user reviews to generate a personal user preferences which can assists in making recommendation in new region.

In this paper, we proposed a location aware POI recommendation system that models user preferences mainly based on user reviews-through which users’ express their sentiments on a particular POI visited hence generally expressing the properties they value and not from certain types of POIs and categories of POIs-which shows the nature of activities that a user finds interesting. Using this information from users’ location history, we predict user ratings by harnessing the information present in review text as well as consider social influence from similar user set formed based on matching category preferences and similar reviews. We use real data sets partitioned by city provided by Yelp, to compare the accuracy of our proposed method against some baseline POI recommendation algorithms.

The following is organized as: Background and related works are discussed in Section 2; while the mortification and objective is analyzed in Section 3; the New Geographical Region POI Recommendation Algorithm is descried in detail in Section 4; Experiments are discussed and explained in Section 5; finally we give conclusion in Section 6.

## 2. Background and Related Works

### 2.1. Local Based Social Networks (LBSNs)

In real physical world, a social network is a social structure that consists of people connected by one or more interdependency such as friendship, common interests and activities, and shared knowledge. In recent years, this structure has been implemented on the Internet and is the core of Online Social Networks (OSNs) that provide social networking services. The increased availability of location-acquisition technologies in mobile devices (e.g., location sensors, GPS and Wi-Fi) has enabled traditional OSNs service providers to incorporate geographical location as a new dimension to the existing OSNs framework. Combined with geographical information, online social networks have evolved to location-based social networks (LBSNs).

**Figure 1:**
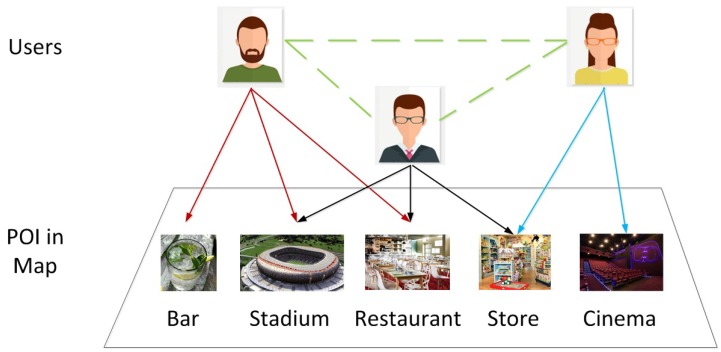
Concept of Location-Based Social Network.

Figure 1 shows the concept of a Location-Based Social Network. As can be observed from the figure in addition to the social networking structure between users, they can share their footprints, opinions, experiences and contribute assorted forms of location-specific multimedia contents in a LBSNs through an action called “check-in” at a point of interest(POI) using their mobile devices.

According to a survey [9], existing LBSNs can be classified into three major groups:

1. Geo-tagged-media-based. Geo-tagging services enable users to label the media content such as text, photos, and videos generated in the physical world.
2. Point-location-based. Applications that encourage people to share their current locations, such as restaurants or museums, which are the most popular type of location-based social networking services.
3. Trajectory-based. In a trajectory-based social networking service is a new type of location-based social networking services.

Therefore, studying a user’s activities history will show various interactions such as between users and also between a user and different location (POIs) and location specific multimedia content tagged at that location by a user. This presents a bridge between the physical world (the POIs) and the virtual social online network interactions between users setting the general context of OSNs in a geographical region or context. In this paper, we focused on Point-location-based location-based social network.

### 2.2. Recommendation System

Recommendation systems (RSs) are software tools and techniques that provide suggestions for items that may be useful to a users’ [10,11]. These suggestions relate to decision making process across different domains, for example, what movie to watch, what music to listen to, which product to buy and where to go out for dinner. Thus, the term “item” suggestion here refers to what the system recommends to a user’s within a particular domain. These systems are primarily designed to focus on a specific type of item ensuring that the core recommendation technique used to generate the recommendations are all customized to provide useful and effective suggestions for that specific type of item. RSs are primarily directed towards individuals who lack sufficient personal experience or competence to evaluate the potentially overwhelming number of alternative items to choose from [12].

Previously, research about personalization and recommendation mechanisms have been mainly proposed in the multimedia domain [13,14,15,16]. However, in the context of this paper, items are Point-Of-Interest (POI) and we choose from all the available POI in a given geographical region (i.e., city) provided by Location-Based Social Networks(LBSNs). Therefore we study application of recommendation system in LBSNs.

The recommendation problem can be defined as estimating the response of a user for new items, based on historical information stored in the system, and suggesting to this user novel and original items for which the predicted response is high. In LBSNs many researchers have applied different types of recommendation techniques to making recommendations, these can be categorized as follows: Collaborative Filtering, Content-Based, Link Analysis and Hybrid based techniques.

Much research has been reported on this domain. In [17] they used the content-based approach in LBSNs by matching user’s profile data including age, gender, cuisine preferences, and income, against the price and category of a restaurant using a Bayesian network model. In [18] the author extends the HITS algorithm for discovering experienced users and interesting locations in an LBSN. In their system, each location is assigned a popularity score, and each user is assigned a hub score, which indicates their travel expertise. Based on a mutually reinforcing relationship, a ranking of expert users and interesting locations is computed. Similarly, [19] extends a random walk-based link analysis algorithm to provide location recommendation.

### 2.3. Personalized POI Recommendation

Ye et al. [20] introduced POI recommendation into LBSNs. Due to the strong correlations between geographical distance and social connections discovered in previous work [2,21], current work on POI recommendation on LBSNs mainly focuses on leveraging the geographical and social properties to improve recommendation effectiveness. Techniques of personalized POI recommendation with geographical influence and social connections mainly study these two elements separately, and then combine their output together with a fused model.

The social influence is usually modeled through friend-based collaborative filtering with either memory-based approaches [4,22,23] or model-based approaches [24]. Ye et al. [25] investigated the geographical influence with a power-law distribution. The hypothesis is that users tend to visit places in short distance. Cho et al. [24] and Cheng [1] investigated the geographical influence through a multi-center Gaussian model. Zhang et al. [26] proposed a Kernel density estimation method to model the geographical influence without knowing a specific type of distribution. Most Recently, Zhang et al. [7] modified this kernel density approach by using a dynamic bandwidth function based on the individual user’s historical data noting the difference in physical coverage between users. All these work further combine the geographical influence with social influence through a fused model based on the sum rule or the product rule for POI recommendation.

There are also work using joint model to study geographical influence and social connections for personalized POI recommendation. Ying et al. [27] proposed a set of features related to social factor, individual preference, and location popularity, and used a regression-tree model to recommend POIs. Gao et al. [28] studied the two factors as a component, named as geo-social correlations, to solve the POI recommendation problem on LBSNs.

Among the current work on LBSNs, temporal information has also attracted much attention from researchers. Ye et al. [29] introduced temporal dimension of daily and weekly check-ins to identify the types of unknown geographic target on LBSNs. Cheng et al. [30] introduced the task of successive personalized POI recommendation in LBSNs by embedding the temporal chronological patterns and localized regions into a matrix factorization method. Yuan et al. [31] incorporated both temporal cyclic information and geographical information through a unified framework for time-aware POI recommendation. Gao et al. [32] discovered the distribution of cyclic patterns and proposed a Gaussian Mixture model for personalized POI recommendation.

Most recently, researchers have started exploring the content information on LBSNs for POI recommendation. Current work of content-aware POI recommendation focuses on one of the three types of content information, i.e., POI-property content, user-interest content, and user sentiment indications. Yang et al. [33] introduced sentiment information into POI recommendation and reported its better performance over state-of-the-art approaches. Hu et al. [34] investigated the user-interest content from Twitter and Yelp, and proposed a topic model for POI recommendation considering both the spatial aspect and textual aspect of user posts. Liu et al. [35] studied the effect of POI-associated tags for POI recommendation with an aggregated LDA model and matrix factorization method. Hu et al. [36] incorporated content information into social correlations and proposed a topic model for POI recommendation. Yin et al. [37] investigated both personal interest and local preference in terms of item-based content on LBSNs and EBSNs. All of this work focuses on one type of the content information without considering the other two and their correlations.

## 3. Motivation and Objective

**Figure 2:**
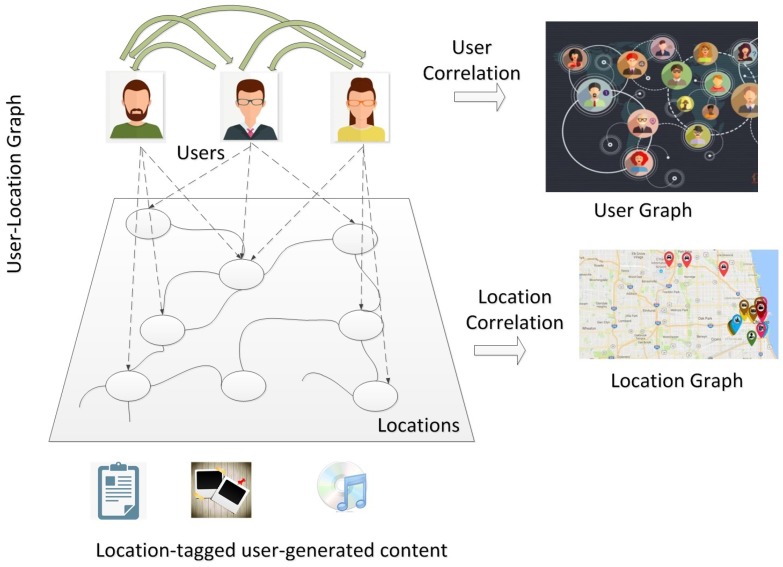
Three relationships derived from LBSNs.

Location-Based Social Networks add a layer of geographical dimension thus we can extract three types of relationship in its structure to form a foundation for our analysis and understand of user behavior, preferences and location properties. Figure 2 [9] highlights three main types of relationships that can be extracted in LBSNs.

As shown in the figure we can extract three layer in a given location-based social network:

1. POI to POI relationship: describing the correlation between the locations based on location similarity according to categories as well as the geo-content tagged such as reviews, shared photo, etc., at these locations by users.
2. Social Links (user relationships): describing the social links or relationship in virtual social network between users usually may explicitly exists as social network friends or can be linked due to similarity of overlap in activity history and POI categories of interest.
3. User Location relationship: describing user to location interaction showing the user’s travel histories, and the strength of this relationship can indicate the number of visits or the user’s review ratings. Based on this information we therefore need to study and exploit these types of relationships in order to make relevant personalized POI suggestion.

From these given relationships we can formally describe properties of LBSNs that form a basis for analysis or incorporation in recommendation models:

1. Social Influence: As LBSNs acts as a bridge between the physical world and the virtual world consistent patterns are expect between social network friends just as it happens in the physical world. For example, friends often go to the same places like restaurants or movie theatres together or a person may travel to a place highly recommended by friends. Thus, a person’s preference on POIs can be influenced by her close friends or a group of friends that are likely to share some common interests. Accordingly, in LBSNs, users establish social links and form communities to share their experiences of visiting POIs. It is importance to note that social influence and geographical properties have a high correlation in LBSNs as friends in different cities will not share a string influence on each other because they will definitely visit different locations.
2. Categorical Influence: In LBSNs POIs possess category information which shows its activities and nature. Therefore by looking at user location history we can get an understanding of the user preferences as certain users have a tendency of visiting particular categories of POI in the cities. For example, one user may have the most check-ins in the arts and entertainment category while others users at restaurants and bars. Additionally, the strength of this relationship will be supported by frequency of the user checking in a particular category or the rating given by this user. Category information can help us find similarities between POIs.
3. Geographic and Temporal Dynamics Influence: As highlighted earlier LBSNs reflect activities in the physical world thus geographical influence affects a user’s check-in behavior as it is have been shown that user tend to visit POIs close to their homes or offices as well as explore places within their reach. Temporal dynamics such as time also affects a user’s check in behavior as a user will check in to a particular category such as a restaurants during noon or evening and arts and entertainment categories in mornings and afternoons or weekends.
4. Tips and Reviews: Content information on LBSNs could be related to a user’s check-in action, providing a unique opportunity for POI recommendation. When checking-in at a POI, a user may leave tips or comments to express his attitude towards the POI. Such content indicates abundant information w.r.t. the user’s interested topics and personal preferences against various facets of the POI. For example, by observing a user’s comment on a Mexican restaurant discussing its spicy food, we observe the User Interests in spicy food. If the comment is actually a compliment, e.g., “Best spicy food ever”, we could infer both the user’s Sentiment Indications and her interests.

This four major features of LBSNs are richly made available from the community contributed data. Therefore, analyzing and applying this information in our approach to making location-based POI recommendation from the many available venues in a huge city in a way that personally targets a user will greatly aid a user in finding an interesting POI.

As we filter POI to provide better matches for users from the largely available POIs unique properties of location-based social networks comes with three major challenges. These are location context awareness, heterogeneous domain and rate at which data grows. In this paper we focus on location context awareness. Geographical dimension in LBSNs bridges the gap between the physical world and the virtual online world. Therefore, the user’s current location is very important in making suggestions, as people are mobile individuals, so it is critical that we provide suggestions within a user’s current geographical area. Hence, in as much as the aforementioned recommendation approach yields accurate results this challenges is non-trivial and may lead to out of context suggestion. Indeed, a location possess a spatial constraint for providing POI recommendations, at the same time influences user preferences. Nevertheless, if a user travels to a new geographical region this becomes even more challenging because a user will have little to no historical information in this geographical area. In this case, the recommendation system faces a cold start problem on such users even though a user has location history from other geographical regions.

A Cold start problem occurs when the recommendation system encounter some individual users with very little history or activity. For the new user, the recommendation model does not have enough knowledge to provide effective suggestion. This being the case with a user in a new region with no social friends (social links) or location history in this region. This is because the new geographical region will different POIs and landscape such that the previous experiences obtain cannot explicitly interpret a user’s local preference in the new region. In addition, social links which have been based on POIs overlaps or friendship may not apply as a particular user is not guaranteed overlapping links in this new environment. For example, beaches might be given a high recommendation score to a user traveling to seaside city, even though the user prefers sporting events more than beaches typically. The same user may be more interested in seeing the status of her friends living in this city. Thus, it is much better to devise a new and sophisticated method to exploit the characteristics of LBSNs, social influence, category, temporal dynamics and user reviews to generate a personal user preferences which can assists in making recommendation in new region.

In view of the foregoing sections, in this paper we set our scope of POI recommendation to consider active LBSNs users’ with a medium to high location activity history traveling to a new geographical region where they have little history. To improve the quality of POI recommendations for this type of users, this paper sets the followings objectives:

1. To model users’ interests and preference learned from users’ activity history in the LBSN such that when a user moves to new geographical area we can still effectively use this information in making new recommendation.
2. To investigate whether similarity computation based on user interest (i.e., reviews and category of choice) provides more quality social opinion than traditional techniques in recommendation irrespective of user’s current location.
3. To exploit and incorporate POI properties and popularity score in order to enhance the quality of the recommendation.

## 4. New Geographical Region POI Recommendation Algorithm Under Big Data Scenario

People are usually active only within small geographical regions within their home city. While it is easy to associate users when they visit similar sets of venues in the same geographical regions, it is interesting and challenging to investigate ways to correlate users across different geographical regions based on their local behaviors. Based on our study, in which we set to understand user behavior across different geographical regions and other patterns that are consistent across regions, we incorporate the studied characteristics in a standard latent factor model because of its ability to incorporate addition information to better describe users’ preferences and items properties in the latent space which it projects for comparison. In the following section we formally introduce the problem we wish to address and describe the framework we propose.

### 4.1. Problem Definition

Suppose that there are *I* users $U^{G_{g}}=\left\{u_{1},u_{2}\ldots u_{I}\right\}$ and *J* POIs $P^{G_{g}}=\left\{p_{1},p_{2}\ldots p_{J}\right\}$ in a given geographical area $G_{g}$, where $G_{g}\in G:\left\{G_{1},G_{2}\ldots G_{g}\right\}$ as set of geographical regions. Let $R^{G_{g}}\in R^{IxJ}$ denote a rating matrix from region $G_{g}$, where $R_{i,j}$ indicates rating of user *i* on $POI_{j}$ and zero is unknown or not rated. In LBSNs user are connected to each other explicitly (i.e., Friendship) or implicitly through a similarity function creating similar user neighborhoods. For this $S^{G_{g}}\in R^{IxI}$ denoting a user relationship (similarity in our cases) matrix where $S_{i,k}$ represents the strength of the relationship between [0, 1]. Users write reviews upon giving a POI a rating and thus each rating user is associated with a set of reviews. For this content we denote a $D^{G_{g}}\in R^{IxW}$ given a vocabulary of words *W* where $D_{i,w}$ represents the importance of a word w∈W is to user *i* based on how often the user uses this word in expressing their preferences. In matrix *D* we treat each user as a document and its content as all words that they have ever written that appear in vocabulary. Given $U^{G_{g}},P^{G_{g}},R^{G_{g}},S^{G_{g}},D^{G_{g}}$ where *g* = 1, 2, 4... We aim to make personalized recommendation of locally interesting $POI\{p_{1},p_{2}\ldots p_{J}\}$ in a region $G_{a}$ to users $\left\{u_{1},u_{2}\ldots u_{I}\right\}$ from a region $G_{b}$ when they migrate to or visit region $G_{a}$ where *a* is geographically different from *b*.

### 4.2. Model Overview

**Figure 3:**
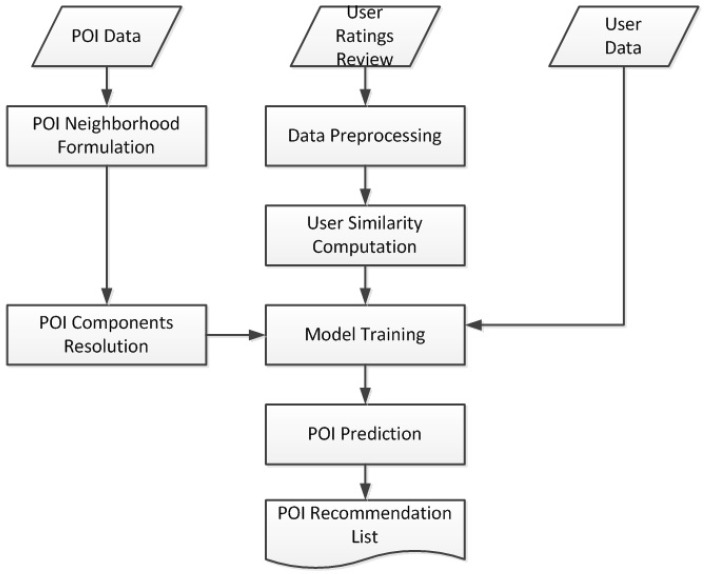
Overview of our recommendation model.

Figure 3 gives an overview of our recommendation model. This model highlights the key features of the recommendation engine which support the proposed model:

1. User Ratings Reviews: This is the input data that contains user rating information and their corresponding reviews. For example a single data point will have user_id, item_id, rating, reviews, date.
2. POI data: This is input data which contains the POI information such as geographical coordinates, category information, etc.
3. User data: This is input data which contains the user related information such as user_id, name, age, friends, registration date, etc.
4. Data preprocessing: This process involve preprocessing of user reviews by concatenating a single user’s entire review text into one collection. This is because we treat a user review set as document such that each user is represented as a document of review in our model’s text analysis component. Prior to building we clean the text, remove stop words, stem each word to their root and finally build a bag of words (vocabulary for our system).
5. User Similarity computation: This takes in user review and user category information. It involves the construction of user to user relationships which forms a basis for building the user similarity neighborhood which is used for social influence contribution to our recommendation model.
6. POI neighborhood: This phase takes in POI data as information required to determine a POI geographical neighborhood that affects its extrinsic characteristics. This neighborhood is required to build the POI neighborhood consider in the recommendation model.
7. POI component resolution: This phase requires POI input data and a POI geographical neighborhood set. It involves resolving the POI into its category latent factors and geoneighbors latent factors as a form of its intrinsic and extrinsic characteristics respectively.
8. Model Training: This is phase in which the model is trained and optimized by incorporating ratings, reviews, social relation and POI information to reach local optimum at which all recommendation model parameters are in a state to give the best recommendation.

### 4.3. Proposed Framework

#### 4.3.1. Basic Model: Latent Factor Model

The baseline rating prediction model used in our framework is a latent factor model based on matrix factorization. Matrix factorization approach is used because they have proven to be accurate, scalable and are very flexible to add auxiliary data sources for recommender such as reviews content and social relations [35]. This implies that our basic recommendation is as follows:

(1) rec(u,p)=α+βi+βj+Ui·Pj

where α is the global offset (average across the dataset), βi and βj are user and POI biases and $Ui=u_{1},u_{2}\ldots u_{i}\in R^{IxF}$ and $Pj=p_{1},p_{2}\ldots p_{J}\in R^{FxJ}$ are F-dimensional user and POI factors respectively. In this basic model, we consider Ui as user preferences towards POIs and Pj as POI properties hence the dot product Ui·Pj matches the interaction between a user and POI. This gives us the following optimization problem:

(2) $$\min_{U*,P*,\beta *}\sum_{(i,j)\in R}{\left(R_{i,j}-rec\left(i,j\right)\right)}^{2}+\lambda_{1}\left({∥Ui∥}^{2}+{∥Pj∥}^{2}+{\beta i}^{2}+{\beta_{j}}^{2}\right)$$

where *i* and *j* have a nonzero $R_{i,j}$ rating in Rating matrix *R* and $\lambda_{1}$ are weights that control the capability of U,P,β in order to avoid over-fitting at which we use Ui,Pj,βiandβj as smoothness regularization terms. According to this basic model it shows that a user traveling to a different region will get a prediction of a locally interesting POI influenced by the global offset and the user’s and item’s rating bias mainly. Indeed, we need to add more user information to better understand this type of user in order to produce a more accurate result.

#### 4.3.2. Integrating Rating with Review: Latent Dirichlet Allocation

Latent Dirichlet Allocation (LDA) uncovers hidden dimensions in review text from which characteristics such as categories, quality and services of a POI reviewed by users can be deduced or a interests in certain categories and characteristics for example ambience, music, menu of restaurant can be deduced. Consequently, we can state that reviews explain the ratings of users, thus help to understand the rating behavior of users, and alleviate the cold-start problem, in this case, where a user moves to a new geographical region where a user has no historical information. By considering each user as document and their reviews as content we can extend our model to capture a user’s preferences. We add an LDA component to our basic model as a regularizer so as to control Ui (user latent vector) by giving us more information about the user. Therefore our optimization problem changes to the following:

(3) $$\min_{U*,P*,\beta *}\sum_{(i,j)\in R}{\left(R_{i,j}-rec\left(i,j\right)\right)}^{2}+\lambda_{rev}\sum_{u\in D}^{I}\sum_{n\in Vd}\log\theta_{z_{u,n}}\cdot \phi_{z_{u,n},w_{u,n}}+\lambda_{1}\left(R\right)$$

where LDA parameters θ and ϕ denote the topic and word distributions, respectively; $w_{u,n}$ and $z_{u,n}$ are the $n^{th}$ word occurring in user *u* and the corresponding topic and λ control contribution of the LDA regularization term addition the effect of the user review. Note that *R* represents the regularization terms ${∥Ui∥}^{2}+{∥Pj∥}^{2}+{\beta_{i}}^{2}+\beta_{j}^{2}$. One thing to note, however, is in order to achieve this, both modeling ratings accurately and generating reviews are likely. The idea of fusing ratings and reviews is through the transformation.

(4) $$\theta_{i,f}=\frac{exp(\kappa U_{i,f})}{\sum_{f}exp\left(\kappa U_{i,f}\right)}$$

where the parameter κ is used to control the quality of the transformation being peaky and $\sum_{f}$ is the summation across each latent topics *f*. In this transformation we expect that the real valued parameters in the user preference vector Ui associated with ratings to be transformed to the probabilistic ones $\theta_{i}$ associated with the reviews. This works because if a POI exhibits a certain property, it will correspond to some topics being commented by users. We adopt HFT algorithm as a component of our proposed system [38].

#### 4.3.3. Integrating Social Influence

Most LBSNs recommendation systems only consider direct friendships or users with physically overlapping visits to POI as a basis for social influence to improve accuracy. However, they are less effective when a targeted user has very few social connection or location history. Similarly, in our case, such an approach would be ineffective because when a user moves a new geographical region they may be the first in their friendship circle to visit this region and will definitely have few visits.

According to our analysis, we observed that users across different geographical regions exhibit similar patterns or preferences. In addition, we found that a user’s behavior in a new region does not drift further apart from the behavior they exhibited in the old region based on category preferences and topics discovers from their ratings and reviews.

**Figure 4:**
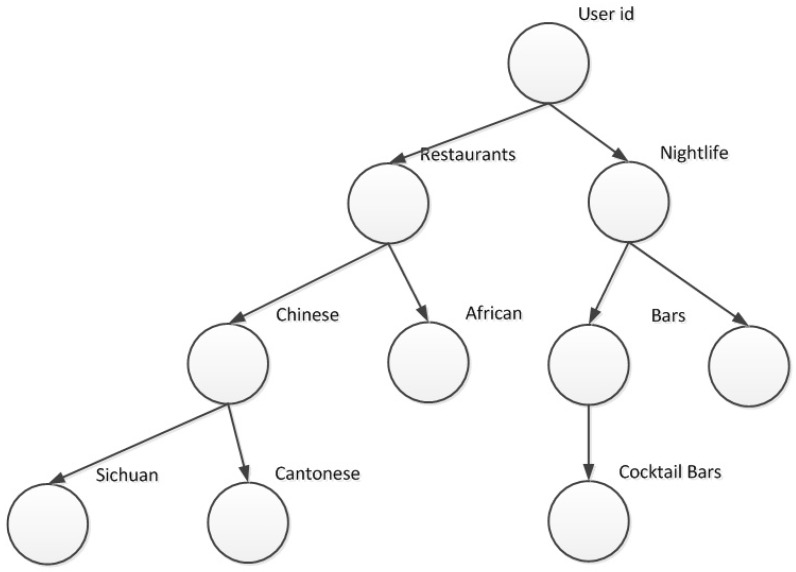
WCH for user.

We use a weighted hierarchical category approach to model user preferences in order to form a basis for similarity comparison between users irrespective of their geographical region in order that the discovered user neighbors will be able to provide social opinion. Firstly, users’ preferences are extracted from users’ historical activity information from all regions G by grouping them according to category forming a three level tree, viz., primary category (level 1), secondary category (level 2) and tertiary category (level 3) as shown in Figure 4. This structure is motivated by our findings in our analysis in which we observed that a user tends to visits POIs of certain types of categories and this pattern showed some similarity where the user moved to a different POI. Furthermore, category interest exists at different granularities; for example, a user will be interested in Restaurants ⟶ Chinese food ⟶ Sichuan noodles.

The nodes of the user WCH (Weighted Category Hierarchical) graph is calculated using term frequency-inverse document frequency (i.e., is a numerical statistic that is intended to reflect how important a word is to a document in a collection) in which each user’s history is treated as a document and categories as terms. We use Equation (5) below:

(5) $$nodeWeight\left(u,c^{\prime }\right)=\frac{\left|\{u.vi:vi.c=c^{\prime }\}\right|}{u.V}\times lg\frac{\left|U\right|}{\left|\{uj:c^{\prime }\in uj.C\}\right|}$$

where $\left|\{u.vi:vi.c=c^{\prime }\}\right|$ is user $u^{\prime }$s number of visits in category $c^{\prime }$, u.V is the total number of the user’s visits, and $\left|\{uj:c^{\prime }\in uj.C\}\right|$ counts the number of users who have visited category $c^{\prime }$ among all the users *U* in the system. This representation of user preference is not tied to users’ location it simply reflects user activities or interest based on historical check-ins as such the structure goes with the users at any location new or old reducing sparsity problem in new location but also help fetch similar users effectively.

User similarity is computed according to the users’ WCH graphs depending on where the user is currently located or specified. Since a WCH is essentially a tree, we measure the similarity between the two WCHs in terms of both their structures and the preference weights associated with each overlapped node. Specifically, we decompose the similarity between two WCHs as a weighted sum of the similarities between each corresponding level of the WCHs. The deeper levels are given a bigger weight as they represent a finer granularity of an individual’s preferences. The approach takes a step by step approach by computing similarity at each level by looking at the number of overlapped categories. Consequently, we use Equation (6) to calculate the similarity as follows:

(6) $$CSim\left(u,u^{\prime }\right)=\sum_{l=1}^{3}\beta_{w}\times \frac{levelSim\left(\right)u,u^{\prime },l}{1+|entr\left(u,l\right)-entr\left(u^{\prime },l\right)|}$$

where $u,u^{\prime }$ are two users in same geographical area and *l* is the number of levels, $\beta_{w}$ is the weight at that level, and $levelSim(u,u^{\prime },l)$ and entr(u,l) are Equation (7) and Equation (8) described as;

(7) $$LevelSim\left(u,u^{\prime },l\right)=\sum_{c\in C^{l}}min\left(u.wc,u^{\prime }.wc\right)$$

where $c\in C^{l}$ is category at a certain level, u.wc and $u^{\prime }.wc$ are two users category at a given level. This function returns the minimum value of the two weights implying that if both users share a high interest in that category we would get a high value and if they do not agree in this category the lowest value affects the overall similarity. Equation (8) below manages the entropy of a user category such that it gives the important or probability of a user visiting the given category according to the historical data. This helps determine the diversity of a user thus measuring the relative of focus of two users across overlapping categories.

(8) $$entr\left(u,l\right)=\sum_{c\in C^{l}}u.P\left(c\right)\times log\;u.P\left(c\right)$$

where P(c) is the probability of user *u* visiting category *c*.

Some observations made in our data analysis shows that from user reviews we can have a more descriptive understanding of a user’s preference through topic modeling approaches. However, only considering review as a basis for comparison between users might not give accurate results as a user may express similar sentiments towards different types of POI. Similarly, user may visit the same POI belonging to a particular category targeting different properties at that POI. Therefore, incorporating user reviews into our similarity computation contributes to a more concise matching between a set of users.

Firstly, we use Latent Dirichlet Allocation Model to discover users-to-words interaction by treating each user as a documents with mixture of latent topics (interest) and latent topics as probability distribution over users’ review words. We achieve the optimal solution using the following Gibbs sampling by iteratively passing through each word to compute the probability of that word in a document to a topic and updating its status to that topic as well as that topics probability to document. Equation (9) shows computation of posterior distribution over latent topic *z*. It should be noted that prior to sampling each unique occurrence of a word in a user document of reviews is assigned an initial topic.

(9) $$\rho \left(z_{i}=j|z_{-i},w_{i},u_{i}\right)={\phi^{\prime }}_{w_{i}}^{\left(j\right)}\cdot {\theta^{\prime }}_{j}^{\left(u\right)}$$

where *j* is the current topic, that we compute the probability that *j* is chosen for word $w_{i}$, the current word being sampled, conditioned on all other assigned topics of words in this user document $u_{i}$ and all other observed variables. Equations (10) and (11) compute ${\phi^{\prime }}^{\left(j\right)}$ latent topics distribution over words and ${\theta^{\prime }}^{\left(u\right)}$ represent the latent topic probability distribution for a user respectively.

(10) $${\phi^{\prime }}_{i}^{\left(j\right)}=\frac{C_{w_{i,j}}^{WT}+\beta_{r}}{\sum_{w=1}^{W}C_{w_{j}}^{WT}+W\beta_{r}}$$

where $C_{w_{i,j}}^{WT}$ count number of times a word token $w_{i}$ was assigned to a topic *j* across all user docs. *W* is the total number of unique words in the corpus (dictionary) and $\beta_{r}$ denotes the relative strength of word combinations in latent topics.

(11) $${\theta^{\prime }}_{j}^{\left(u\right)}=\frac{C_{u_{i,j}}^{UT}+\alpha_{r}}{\sum_{t=1}^{T}C_{u_{i,t}}^{UT}+T\alpha_{r}}$$

where $C_{u_{i,j}}^{UT}$ count number of times a topic *j* was already assigned to some word token in user doc $u_{i}$. *T* is the total number of topics and $\alpha_{r}$ denotes the relative strength of latent hidden topics in the document. When we obtain our optimal solution we use the variable θ representing the document to topic matrix containing the document distributions over words.

We then use cosine similarity to find the similarity between any two users *i* and *k* using their probability distribution across latent topics given by $\theta^{\left(u_{i}\right)}$ and $\theta^{\left(u_{k}\right)}$ as follows:

(12) $$Rsim\left(i,k\right)=\frac{\theta^{\left(u_{i}\right)}\theta^{\left(u_{k}\right)}}{∥\theta^{\left(u_{i}\right)}∥\cdot ∥\theta^{\left(u_{k}\right)}∥}$$

where Rsim(i,k) is the similarity value calculated from user latent topic vectors $\theta^{\left(u_{i}\right)}$ and $\theta^{\left(u_{k}\right)}$. Thus Equation (13) shows the addition of this similarity value to extend the category-based similarity.

(13) Sim(i,k)=μCSim(i,k)+(1−μ)Rsim(i,k)

where CSim(i,k) is the category-based similarity and Rsim(i,k) is review-based similarity. The μ controls the contribution of the review information to the category-based similarity.

Using this similarity information we build our similarity matrix $S\in R^{\left(IxI\right)}$ containing $S_{i,k}$ similarity between any two users *i* and *k*. This matrix will assist us in providing additional information for building a neighborhood of similar users to offer social influence hence local opinion in a new geographical region unfamiliar to the user. Equation (14) shows how we integrate Social Influence in our Recommender Systems to give us the final objective function.

(14) $$\begin{array}{c} \min_{U*,P*,\beta *}\sum_{(i,j)\in R}{\left(R_{i,j}-rec\left(i,j\right)\right)}^{2}+\lambda_{rev}\sum_{u\in D}^{I}\sum_{n\in Vd}\log\theta_{z_{u,n}}\cdot \phi_{z_{u,n},w_{u,n}}\\ +\lambda_{rel}\sum_{S_{i,k}\neq 0}\left(S_{i,k}-U_{I}^{T}HU_{k}\right)+\lambda_{1}\left(R\right)+\lambda_{2}\left(∥H∥\right) \end{array}$$

where $S_{i,k}$ is the similarity between two user *i* and *k*, *U* is the user vector from the user latent factor matrix and *H* is the social correlation matrix. $\lambda_{rel}\;,\;\lambda_{2}$ and *H* are introduce as weights to control the contribution of the social correlation and over-fitting respectively.

#### 4.3.4. Integrating POI Characteristics

In LBSNs a POI characteristics affects its rating. Therefore the more information we have about a POI the more accurate a recommendation of a particular POI. In this study POI we assume that a POI has two types of characteristic set, intrinsic properties such as the surrounding environment, and the intrinsic properties such as the quality of product and service. Consequently, we assume that a user’s rating of a given business is determined by its intrinsic characteristics and the extrinsic characteristics of its geographical neighbors. Therefore we divide the POI into latent factors of extrinsic properties $Q\in R^{F}$ its geographical neighbors and latent factor of intrinsic properties $D\in R^{F}$ its categories. It should be noted however that more intrinsic and extrinsic properties can be added in order to capture more aspects of POI hence more accurate results.

According to our observations, most businesses have neighbors within a short geographical distance, and more importantly, the rating of a business is weakly positively correlated with the rating of its neighbors. These observations suggest that considering the geographical neighborhood influence may improve the accuracy of business rating prediction. Let $N_{i}$ be a set of geographical neighbors for a business *i*, satisfying certain criterion selection (e.g., the top-10 nearest neighbors). Let $n\in N_{i}$ be neighbors of business *i*. This gives us our new prediction ratings computation as shown in Equation (15):

(15) $$rec\left(u,p\right)=\alpha +\beta_{i}+\beta_{j}+U_{i}\cdot \left(P_{j}+\frac{\alpha_{1}}{|N_{j}|}\cdot \sum_{n\in N_{j}}Q_{n}\right)$$

In the above equation, parameter $\alpha_{1}$ is a weight that controls the importance of the influence of the geographical neighborhood and $|N_{j}|$ denoting the cardinality of the set of neighbors. $Q_{n}$ is the latent factor component that models the extrinsic characteristics of the POI. Extending our Objective function we add $Q_{n}$ as a regularization terms.

(16) $$\begin{array}{c} \min_{U*,P*,\beta *}\sum_{(i,j)\in R}{\left(R_{i,j}-rec\left(i,j\right)\right)}^{2}+\lambda_{rev}\sum_{u\in D}^{I}\sum_{n\in Vd}\log\theta_{z_{u,n}}\cdot \phi_{z_{u,n},w_{u,n}}\\ +\lambda_{rel}\sum_{S_{i,k}\neq 0}\left(S_{i,k}-U_{I}^{T}HU_{k}\right)+\lambda_{1}(R+\sum_{n\in N_{j}}∥Q_{n}{∥}^{2})+\lambda_{2}\left(∥H∥\right) \end{array}$$

The category of a POI is important because it gives an indication of the services or activities that take place or the way the business is conducted at a POI. Furthermore, users show more interest towards certain categories than others. Consequently, adding categories information to a POI leads to better accuracy [39]. In this study we annotate the POI with a category information by integrating a category latent factor vector $D\in R^{F}$ per category. Implicitly, similar category of POI will tend to influence each other’s rating giving and overview of how the local people feel about these category. Equation (17) shows how the category latent factor vector is integrated into our model.

(17) $$rec\left(u,p\right)=\alpha +\beta_{i}+\beta_{j}+U_{i}\cdot \left(P_{j}+\frac{\alpha_{1}}{|N_{j}|}\cdot \sum_{n\in N_{j}}Q_{n}+\frac{\alpha_{2}}{|D_{j}|}\cdot \sum_{c\in C_{j}}D_{c}\right)$$

In the above equation, parameter $\alpha_{2}$ is a weight that controls the importance of the influence of the geographical neighborhood and $|D_{j}|$ denoting the cardinality of the set of neighbors. $Q_{n}$ is the latent factor component that models the extrinsic characteristics of the POI. Extending our Objective function we add $D_{c}$ as a regularization terms.

(18) $$\begin{array}{c} \min_{U*,P*,\beta *}\sum_{(i,j)\in R}{\left(R_{i,j}-rec\left(i,j\right)\right)}^{2}+\lambda_{rev}\sum_{u\in D}^{I}\sum_{n\in Vd}\log\theta_{z_{u,n}}\cdot \phi_{z_{u,n},w_{u,n}}\\ +\lambda_{rel}\sum_{S_{i,k}\neq 0}\left(S_{i,k}-U_{I}^{T}HU_{k}\right)+\lambda_{1}(R+\sum_{n\in N_{j}}∥Q_{n}{∥}^{2}+\sum_{c\in C_{j}}∥D_{c}{∥}^{2})\\ +\lambda_{2}\left(∥H∥\right) \end{array}$$

How popular a POI is gives a good reflection of the quality of the services or product it offers. Popularity can affect the user check-in behaviors to a great extent. Usually, an individual’s decision to check in a POI is largely affected by the word-of-mouth opinions, which can be represented as the popularity of the POI. However, popularity is based on category and region. For example, POI like restaurants located in downtown area are likely to receive more visits than those in suburban in addition restaurants are more popular than museums. In accordance to recent study [25] we normalize the popularity score in a region level. Equation (19) shows the normalization given number of ratings/visits a particular $POI_{j}$ has received is $totalRv_{j}$ and total users $totalUser_{j}$ given a region *r*.

(19) $$p_{j}=\frac{1}{2}\left\{\frac{totalUser_{j}-1}{\max_{j\in r}\left\{totalUser_{j}-1\right\}}+\frac{totalRv_{j}-1}{\max_{j\in r}\left\{totalRv_{j}-1\right\}}\right\}$$

where $\max_{j\in r}totalUser_{j}-1$ and $\max_{j\in r}totalRv_{j}-1$ are the maximum total number of people and total check-in number in the region respectively. We integrate the normalized popularity in our recommendation model.

(20) $$rec\left(u,p\right)=\alpha +\beta_{i}+\beta_{j}+\gamma_{j}p_{j}U_{i}\cdot \left(P_{j}+\frac{\alpha_{1}}{|N_{j}|}\cdot \sum_{n\in N_{j}}Q_{n}+\frac{\alpha_{2}}{|D_{j}|}\cdot \sum_{c\in C_{j}}D_{c}\right)$$

where $\gamma_{j}$ controls the contribution of the popularity $p_{j}$ to the prediction rating.

#### 4.3.5. Model Training

Finally, our objective function, which we wish to optimize in order to make accurate predictions, is as follows.

(21) $$\begin{array}{c} Y\left(\Theta ,\Phi ,z,\kappa \right)≜\min_{U*,P*,\beta *}\underset{rating\;error}{\underset{︸}{\sum_{(i,j)\in R}{\left(R_{i,j}-rec\left(i,j\right)\right)}^{2}}}+\\ \underset{reviews\;likelihood}{\underset{︸}{\lambda_{rev}\sum_{u\in D}^{I}\sum_{n\in V_{d}}\log\;\theta_{z_{u},n}\phi_{z_{u},w_{u},n}}}+\underset{localsocial\;opinion}{\underset{︸}{\lambda_{rel}\sum_{S_{i,k}\neq 0}\left(S_{i,k}-U_{I}^{T}HU_{k}\right)}}+\underset{regularization}{\underset{︸}{\lambda \Omega (\Theta )}} \end{array}$$

where $argmin_{\Theta ,\Phi ,z,\kappa }Y\left(\Theta ,\Phi ,z,\kappa \right))$ is our objective function which we wish to minimize. Θ represents the parameter set U,P,H,Q,D,γ i.e., the users, POI, social correlation, POI neighbor and category latent factors which are associated with the ratings and social relation and Φ represents the parameters θ,ϕ associated with the review text. Parameter set z,κ are the latent topics and controller for transformation between ratings and reviews. λΩ(Θ) is the regularization terms as follows:

(22) $$\lambda \Omega \left(\Theta \right)=\lambda \left(∥U_{i}{∥}^{2}+∥P_{j}{∥}^{2}+\beta_{i}^{2}+\beta_{j}^{2}+\sum_{n\in N_{j}}∥Q_{n}{∥}^{2}+\sum_{c\in C_{j}}∥D_{c}{∥}^{2}+{∥H∥}^{2}\right)$$

We use stochastic gradient descent approach (GD) to find our optimal solution. Therefore, we determine the gradient of each variable at every iteration in order to update its state by moving it towards the best estimate value as results giving us the overall best estimate for the entire system. The connection between ratings and social influence is the realized through the users latent feature space *U*, and ratings and reviews are linked through the transformation involving *U* and θ through Equation (4). Our objective function optimal solution can be found by gradient descent and the latter by Gibbs sampling.

## 5. Experiments

In this section, we conduct experiments to evaluate the recommendation quality of the proposed recommendation model against some baseline state-of-art POI recommendation techniques.

### 5.1. Experimental Framework

#### 5.1.1. Dataset

We evaluate our models on the yelp dataset, which has more than 10 cities from 4 countries. We list the most active cities and their corresponding user ratings count, viz, Las Vegas, NV-(617,352), Phoenix, AZ-(219,828), Scottsdale, AZ-(120,756), Tempe, AZ-(64,978), Edinburgh, EDH-(22,761), Waterloo, ON-(1680), Montreal, QC-(28,683). However, we found that Phoenix and Las Vegas have a relatively large amount of rating and review data as well as a high number of overlapping users (users with activities in both cities) hence we consider these two cities for our experiments.

In our experiments, we shall consider our target users as the users from phoenix with ratings in Las Vegas and vice versa. It should be noted that for simplicity, we consider a city as our geographical region for testing, however a finer granularity can be considered such as a districts from one city as our algorithms attempts to predict ratings for users who move to a new geographical region. The dataset statistics for the two cities are shown in Table 1. The yelp dataset provided does not explicitly contains a user’s home location or address. Therefore, for experimental purposes we make an assumption that a user’s most active city is the user’s home location. Activity in this case is the total count of ratings and reviews left by a user at POIs in a given city. We use the local ratings/reviews as the training set, including 1–3 foreign reviews for our target users and use the remaining set of foreign ratings and reviews as test data.

#### 5.1.2. Evaluation Metrics

We adopt the Mean Absolute Error (MAE) and normalized MAE (rMAE) to measure the accuracy of predicted ratings which measures the average absolute deviation between a predicted rating and the user’s true ratings. MAE is define as follows:

(23) $$MAE=\frac{1}{\left|N\right|}\sum_{u_{i}p_{j}}\left|{\hat{R}}_{i,j}-R_{i,j}\right|$$

where |N| denotes the number of tested ratings, $R_{\left(i,j\right)}$ is the real ratings, and ${\hat{R}}_{i,j}$ is a predicted ratings. This approach is because the predicted rating values create an ordering across the items, predictive accuracy can also be used to measure the ability of a recommender system to rank items with respect to user preference [40].

#### 5.1.3. Baselines

To evaluate the effectiveness of our proposed solution, we compare it with the following baseline approaches:

1. User-KNN: This method is the user-based collaborative filtering. The unknown ratings are predicted by considering the ratings given by similar users considered as the user’s neighborhood. User similarity is computed by cosine similarity of ratings and we set the neighborhood size k = 150 in our experiments.
2. Item-KNN: This method is the item-based collaborative filtering. The unknown ratings are predicted by considering the ratings received by similar items (item neighborhood). Item similarity is computed by cosine similarity of ratings and we set the neighborhood size k = 150.
3. User Cluster(UC): This is a graphical model that clusters users into K communities(groups) for recommendation [41].
4. CKNN: This projects a user’s activity history into the category space and models user preference using a weighted category hierarchy. When receiving a query, CKNN retrieves all the users and items located in the querying city, formulates a user-item matrix online, and then applies a user-based CF method to predict the querying user rating of an unvisited item. Note that the similarity between two users in CKNN is computed according to their weights in the category hierarchy, making CKNN a hybrid recommendation method [42].
5. SVD++: This model considers implicit feedback from users for rating prediction. SVD++ usually offers a superior accuracy and is considered as a state-of-the-art matrix factorization algorithm for recommendation [43].
6. HFT: This method combines basic latent factors in ratings with hidden topics in reviews [38]. This approach uses ratings and reviews only.
7. NRLRS: We name our algorithm New Region Location Recommender System (NRLRS) because of its proposed ability to alleviate cold -start problem when a user travels to a new geographical region.

We have done preprocessing with the acquired data. All those baseline approaches have been implemented. We divide the pre-processed data into training set and test set according to normal standard. We train those model with training set, and use test set to justify the accuracy of those methods.

### 5.2. Results and Analysis

We use librec1.4 a recommendation system library in java for algorithms implementation and extension [44]. For all the latent factor models we set the default F=10 otherwise stated. We set the learning rate ω=0.0005, momentum = 0.8 and the weights $\lambda_{rel}=0.0025$ and $\lambda_{rev}=0.05$. The results of comparison are presented per city (Phoenix and Las Vegas) and are reported in Table 2 and Table 3.

**Figure 5:**
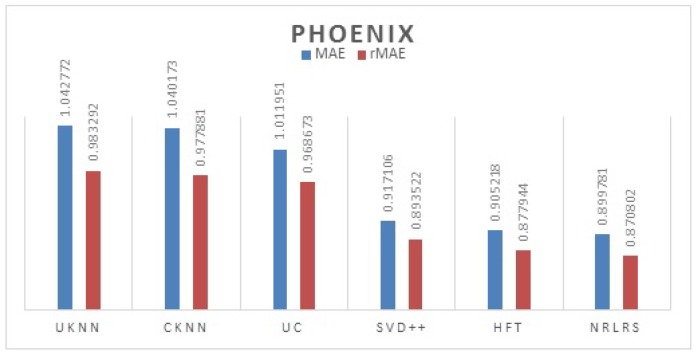
Phoenix Test Results.

**Figure 6:**
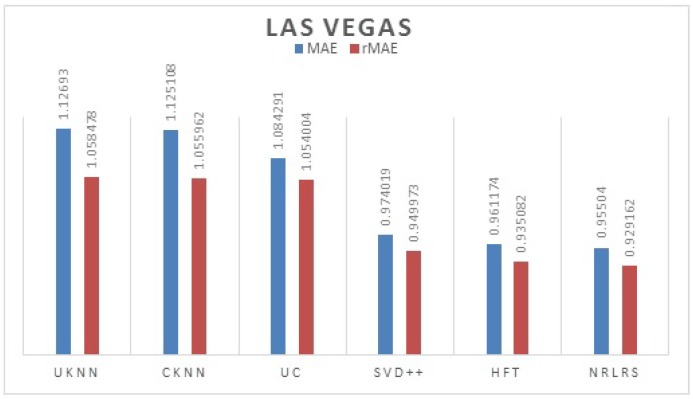
Las Vegas test Results.

We Plot the results MAE and rMAE of each Algorithms in Figure 5 for Phoenix datasets, Figure 6 for Las Vegas datasets.

As can be observed from our results from both cities, the least performing are the neighborhood models UKNN and IKKN. This is expected because when a user moves to a geographical region where they have little or no activity history this presents a problem of limited information to match them with other users. Neighborhood approaches consider overlapping visited POI/items between users to determine similarity in preferences therefore this information is limited for a user with few ratings leading to a cold -start problem. CKNN and User Clusters methods performance is slightly better because in this approaches we incorporate user’s category preferences from previous cities activity history to build a user preferences that assist a user find a group of similar users locally to offer social influence in POI recommendation. The latent factors models all outperform the neighborhood models because of their ability to discover some hidden features between the interaction of the user preferences and the POI properties with addition implicit information. Further we observe that HFT and NRLRS show better accuracy because of their ability to exploit and incorporate an active users reviews from their previous geographical region into the new region providing these algorithms with implicit information for modeling the user preferences better. NRLRS has the best results as it incorporate variety information which helps us model POI properties and user preferences better for a new user in addition to integrating the neighborhood model feature of social influence (local opinion) which is important component for a traveling or migrating user to a new location. Different cities show different prediction accuracy values due to difference in the datasets statistics and patterns specific to individual cities, however the consistency in performance is shown across the different algorithms.

We test for performance of the latent factor models by varying the number of latent factors assigned thus we adjust the number of latent factors and record the prediction accuracy results for each algorithm per city. This is tested with respect to the earlier wisdom that latent factor model tend to use more factors, hence an increase in the factors is expected to show an increment in ratings prediction [45]. In Table 4 and Table 5, we show our results. We use MAE to test the variation of the accuracy with the increase in the number of factors.

**Figure 7:**
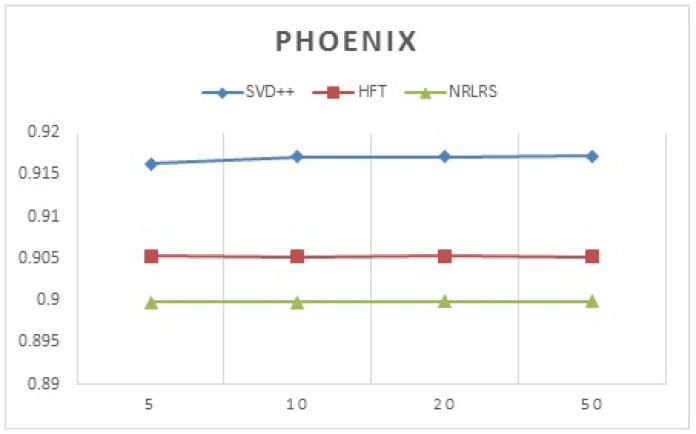
Prediction by varying latent factors (Phoenix).

In Figure 7 we show a plot of the recommendation prediction of all the latent factor models across different latent factors using Phoenix datasets.

**Figure 8:**
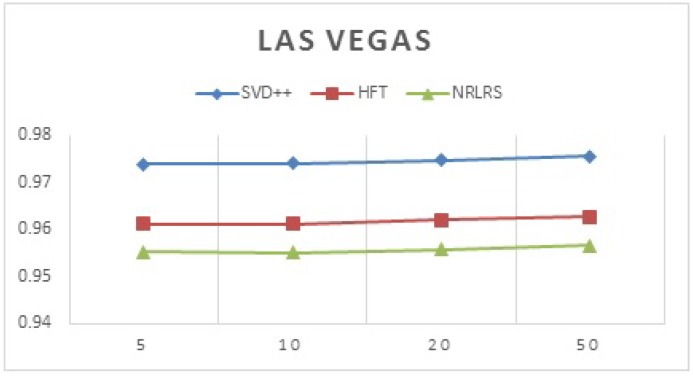
Prediction by varying latent factors (Las Vegas).

Below are the prediction results for latent factor model algorithms with varying factors in Las Vegas (shown in Figure 8).

According to our findings, the accuracy SVD++, HFT and NRLRS do not show much variation with a change in the number of latent factors F and show stability across different size of F. It was found that F = 10 gives approximately the best prediction results as such we adopt it as default for experimental evaluation. We investigate the impact of social local opinion, POI properties and Reviews. We define our algorithms as followings; (1) NRLRS\Rev: Consider all features but the reviews component, we set $\lambda_{rev}=0$; (2) NRLRS\Social: Considers all feature but the Social Relations component, we set $\lambda_{rel}$=0; (3) NRLRS\Social\Rev: Considers features all but the social relation and review features by setting $\lambda_{rev}=0$ and $\lambda_{rel}=0$, and finally (4) NRLRS\POI: Considers all features but the POI properties by setting $\alpha_{1}=0$ and $\alpha_{2}=0$. Table 6 and Table 7, show the report on this investigation.

**Figure 9:**
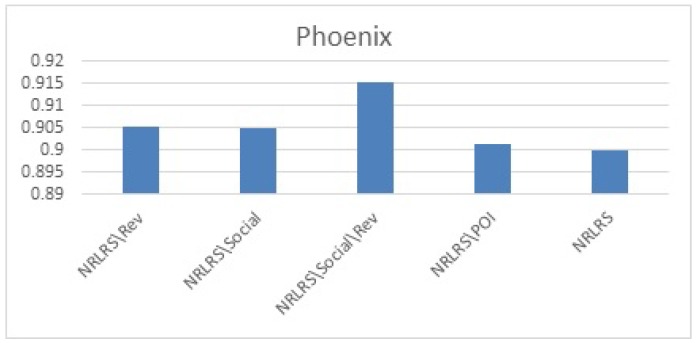
Impact of components Phoenix Results.

**Figure 10:**
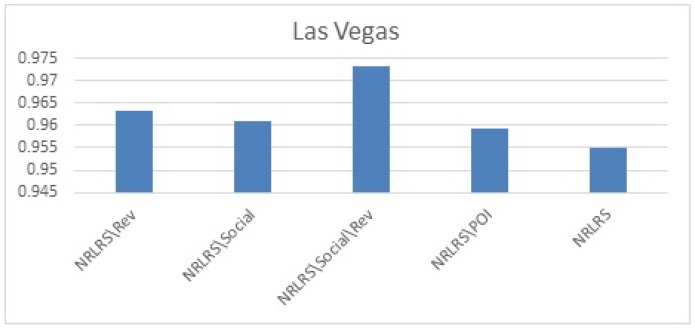
Impact of Components Las Vegas Results.

The results (as also shown in Figure 9 and Figure 10) show that the performance degrades when any of the components is eliminated demonstrating the importance of each components contribution to the entire models. We note that our models performance without our proposed integrated components shows a performance comparable to SVD++ algorithm across the two cities. Furthermore, we note that reviews (NRLRS\Rev) seem to show a very strong contribution given that when we remove reviews the performance of the algorithm significantly degrades. This is in accordance with the understanding that reviews contain a reflection of user preferences or item properties because these are expressed when the users expresses their sentiments in reviews. Therefore, leading to a better insights into user preferences for POI recommendation. Social influence from user groups of similar matching category and reviews also shows a significant contribution owing to the degradation of the prediction error when social influence is remove. POI properties influence does not show a big influence in our prediction accuracy although the improvement it offers is significant and cannot be considered trivial.

In view of the foregoing, in these experiments we demonstrate that our proposed solution performance achieves a higher prediction accuracy in our set context of exclusively considering traveling users to new geographical regions, in the case of our datasets Phoenix users traveling to Las Vegas and vice versa. Although the improvements of MAE we obtain may seem small, they are significant. Koren [43] provides evidence that even a small improvement in a rating prediction error can affect the ordering of items and have significant impact on the quality of the top few presented recommendations and thus the overall performance of the recommender system.

## 6. Conclusions

With the development of wireless Internet and the popularity of location sensors in mobile phones, the coupling degree between social networks and location sensor information is increasing. This paper proposed a point of interest (POI) recommendation algorithm for users who move to any given new geographical region where they have little or no activity history to infer their interests and preferences in location-based social networks (LBSNs) in order to provide quality recommendation under big data scenario. We set to alleviate this lack of sufficient information for building user preferences for a traveling user in a new region but using their reviews and categorical information from their activity history in LBSN from all regions they have visited before, in order to understand their personal interests and preferences which may continue to be applicable in any new region they visit. The complexity and the implementation cost of the proposed framework is comparable with the latent factor model, which is used as the baseline rating prediction model of our framework.

Future works to be considered could be the extension of our model to many more cities in order the study and understand users’ ratings and review behavior as they move between different cities, as this will give us more concrete conclusions. This is because LBSNs connect the virtual online world to the physical world, which makes their study interesting but challenging. Adding, other temporal information such as time-context and other temporal dynamics will contribute to a more enhanced accuracy to the proposed model. Because evaluate a recommender system in the world is challenges due to factors such as novelty building a mobile application to evaluate and survey its performance in a real world instance will provide a more clear understanding.

It is worth mentioning that the problem we study and the method we propose in this paper are applicable to all LBSNs and we chose Yelp for our tests in this work.

## Figures and Tables

**Table 1:** Two most active cities.

Statistics	Phoenix, Arizona	Las Vegas, Nevada
#users with review	65,191	173,703
#Reviews/rating	219,828	617,352
#Businesses	8406	13,592
#users with review	65,191	173,703
#Min/Max review per Business	1/1354	1/4137
#foreign Reviews/ratings	78,948	195,205
#Min/Max review per User	1/607	1/1126

**Table 2:** MAE and rMAE Comparisons of different methods (F = 10, KNN = 150)-Phoenix.

Algorithm	MAE	rMAE
UKNN	1.042772	0.983292
IKNN	1.045428	0.995892
UC	1.011951	0.968673
CKNN	1.040173	0.977881
SVD++	0.917106	0.893522
HFT	0.905218	0.877944
NRLRS	0.899781	0.870802

**Table 3:** MAE and rMAE Comparisons of different methods (F = 10, KNN = 150) -Las Vegas.

Algorithm	MAE	rMAE
UKNN	1.126930	1.058478
IKNN	1.123261	1.061438
UC	1.084291	1.054004
CKNN	1.125108	1.055962
SVD++	0.974019	0.949973
HFT	0.961174	0.935082
NRLRS	0.955040	0.929162

**Table 4:** Prediction Performance by varying number of latent factors (Phoenix).

Factors (K)	SVD++	HFT	NRLRS
5	0.916287	0.905274	0.899798
10	0.917106	0.905218	0.899781
20	0.917182	0.905291	0.899883
50	0.917237	0.905233	0.899907

**Table 5:** Prediction by varying latent Factors (Las Vegas).

Factors (K)	SVD++	HFT	NRLRS
5	0.973807	0.961213	0.955177
10	0.974019	0.961174	0.955040
20	0.974787	0.961932	0.955783
50	0.975496	0.962651	0.956517

**Table 6:** Impact of Components (Phoenix).

Algorithms	NRLRS\Rev	NRLRS\Social	NRLRS\Social\Rev	NRLRS\POI	NRLRS
MAE	0.905218	0.904739	0.915097	0.901132	0.899781

**Table 7:** Impact of Components (Las Vegas).

Algorithms	NRLRS\Rev	NRLRS\Social	NRLRS\Social\Rev	NRLRS\POI	NRLRS
MAE	0.963223	0.961101	0.973211	0.959185	0.955040
